# Explainable Computational Imaging for Precision Oncology: An Interpretable Deep Learning Framework for Bladder Cancer Histopathology Diagnosis

**DOI:** 10.3390/bioengineering13010004

**Published:** 2025-12-21

**Authors:** Abdallah A. Mohamed, Yousry AbdulAzeem, Abdullateef I. Almudaifer, Hanaa ZainEldin, Hossam Magdy Balaha, Mahmoud Badawy, Mostafa A. Elhosseini

**Affiliations:** 1 Department of Information Systems, College of Computer Science and Engineering, Taibah University, Yanbu 46421, Saudi Arabia; aammohamed@taibahu.edu.sa; 2 Mathematics and Computer Science Department, Faculty of Science, Menoufia University, Menoufia 32511, Egypt; 3 School of Computational Sciences and Artificial Intelligence, Zewail City of Science, Technology and Innovation, Giza 12578, Egypt; yousry@mans.edu.eg; 4 Department of Computer Science, College of Computer Science and Engineering, Taibah University, Yanbu 46421, Saudi Arabia; amdefar@taibahu.edu.sa; 5 Computers and Control Systems Engineering Department, Faculty of Engineering, Mansoura University, Mansoura 35516, Egypt; eng_hanaa@mans.edu.eg (H.Z.); hmbala01@louisville.edu (H.M.B.); engbadawy@mans.edu.eg (M.B.); 6 Bioengineering Department, J.B. Speed School of Engineering, University of Louisville, Louisville, KY 40292, USA; 7 Department of Computer Science and Information, Applied College, Taibah University, Medinah 41461, Saudi Arabia

**Keywords:** bladder cancer, histopathology diagnosis, deep learning, You Only Look Once

## Abstract

Bladder cancer represents a significant health problem worldwide, with it being a major cause of death and characterized by frequent recurrences. Effective treatment hinges on early and accurate diagnosis; however, traditional methods are invasive, time-consuming, and subjective. In this research, we propose a transparent deep learning model based on the YOLOv11 structure to not only enhance lesion detection but also give the visual support of the model’s predictions. Five versions of YOLOv11—nano, small, medium, large, and extra large—were trained and tested by us on a comprehensive dataset of hematoxylin and eosin-stained histopathology slides with the inflammation, urothelial cell carcinoma (UCC), and invalid tissue categories. The YOLOv11-large variant turned out to be the best-performing model at the forefront of technology, with an accuracy of 97.09%, precision and recall of 95.47% each, and balanced accuracy of 96.60%. Besides the precision–recall curves (AUPRC: inflammation = 0.935, invalid = 0.852, UCC = 0.958), ROC-AUC curves (overall AUC = 0.972) and risk–coverage analysis (AUC = 0.994) were also used for detailed assessment of the model to confirm its steadiness and trustworthiness. The confusion matrix displayed the highest true positive rates in all classes and a few misclassifications, which mainly happened between inflammation and invalid samples, indicating a possible morphological overlap. Moreover, as supported by a low Expected Calibration Error (ECE), the model was in great calibration. YOLOv11 reaches higher performance while still being computationally efficient by incorporating advanced architectural features like the C3k2 block and C2PSA spatial attention module. This is a step towards the realization of the AI-assisted bladder cancer diagnostic system that is not only reliable and transparent but also scalable, presented in this work.

## 1. Introduction

Bladder cancer (BC), primarily in the form of urothelial cell carcinoma (UCC), is a serious global health concern. It arises from the uncontrolled growth of malignant cells in the bladder lining, ureters, or renal pelvis. Key risk factors include smoking, exposure to certain chemicals, chronic inflammation, and genetic susceptibility. Common symptoms include blood in the urine (hematuria), changes in urination patterns, pelvic discomfort, and, in advanced cases, weight loss and fatigue [1]. Diagnosis typically relies on imaging (CT, MRI), direct visualization via cystoscopy, and biopsy to confirm malignancy. Treatment options range from surgical removal of tumors or the entire bladder, to chemotherapy, immunotherapy, and radiation. Early detection and prompt treatment are vital for improving patient survival and preventing disease progression [2,3,4,5].

Cystitis, or bladder inflammation, is a distinct but often clinically challenging condition. It can be acute or chronic, frequently caused by bacterial infection, but it can also be triggered by chemical irritants, radiation, or foreign bodies. Cystitis symptoms overlap with BC, including lower abdominal pain, urinary urgency/frequency, hematuria, and cloudy or foul-smelling urine. Diagnosis involves urine tests, cystoscopy, and imaging to exclude other causes. Management usually includes antibiotics, pain relief, irrigation, and lifestyle adjustments. Unlike BC, most cases of cystitis are readily treatable [6].

Despite advances, BC remains a significant global public health issue, ranking among the ten most common cancers worldwide and contributing substantially to cancer-related mortality [7]. The prognosis is heavily dependent on early detection; if high-grade tumors are not treated promptly, the 5-year death rate can soar from 23% to 95% once the cancer has spread [8,9]. Even for non-muscle-invasive tumors, the grade at diagnosis is the strongest predictor of outcome. While hematuria is often the first sign, it is not diagnostic on its own. Current diagnostic workflows involve multiple steps (urine cytology, tumor markers, cystoscopy, CT scans, and biopsy), which are often invasive, time-consuming, and subject to variability between pathologists [10]. Consequently, only about 70% of tumors are detected early, highlighting a critical need for more efficient and reliable diagnostic tools.

Artificial Intelligence (AI), particularly deep learning (DL), offers a promising solution for automating and enhancing medical image analysis [11]. DL models, especially Convolutional Neural Networks (CNNs), excel at automatically learning complex features from images, outperforming traditional methods that rely on manual feature engineering [12,13,14]. The You Only Look Once (YOLO) family of algorithms is renowned for its speed and accuracy in object detection [15,16,17]. YOLOv11, a recent iteration, incorporates architectural innovations like the C3k2 block and C2PSA spatial attention module, making it exceptionally well-suited for tasks requiring both precision and computational efficiency [18,19].

This study proposes a novel, interpretable deep learning framework for classifying histopathology slides of bladder lesions into three clinically relevant categories: inflammation, UCC, and invalid/low-quality tissue. We adapt the YOLOv11 architecture for this multi-class classification task, utilizing its powerful feature extraction capabilities. To ensure clinical trust and utility, we integrate EigenCAM, a technique that generates visual explanations for the model’s predictions by highlighting the specific regions of the slide that influenced its decision. Our work addresses several gaps identified in current research:

- Multi-class Classification: Many studies focus on binary classification (e.g., cancer vs. normal). Our model distinguishes between inflammation, UCC, and invalid tissue, which is crucial for clinical triage.
- Interpretability: We provide transparent, visual explanations for predictions, moving beyond “black-box” models.
- Holistic Framework: We present a complete pipeline, from data acquisition and preprocessing to modeling and evaluation.

The primary objective of this study is to evaluate the performance of our YOLOv11-EigenCAM framework against established methods, focusing on diagnostic accuracy, sensitivity, specificity, and model interpretability. By developing a scalable and accurate tool, we aim to support pathologists in their daily workflow, enabling faster, more consistent, and more confident diagnoses. In summary, our contributions are as follows:

- Adaptation of YOLOv11 for Medical Classification: We demonstrate the effectiveness of the YOLOv11 architecture, originally designed for object detection, for the specific task of multi-class histopathology classification.
- Integration of Interpretability: We incorporate EigenCAM to generate Class Activation Maps, providing clinicians with visual evidence for the model’s predictions and fostering trust in its output.
- Comprehensive Diagnostic Pipeline: We develop a complete, end-to-end system that encompasses data preparation, model training, and rigorous performance evaluation, creating a foundation for clinical deployment.
- High Diagnostic Accuracy: Our model achieves state-of-the-art performance, with an accuracy of up to 97.09%, demonstrating its potential for real-world application.

The remainder of this paper is structured as follows: Section 2 reviews related work in AI for bladder cancer diagnosis. Section 3 details our proposed methodology. Section 4 presents the experimental setup and results. Finally, Section 5 summarizes our findings and outlines future directions.

## 2. Related Studies

Deep learning techniques have demonstrated significant potential in enhancing BC diagnosis, thereby providing opportunities for increased accuracy, efficiency, and automation in lesion detection and interpretation. This section reviews the pertinent literature on the application of DL for BC diagnosis across different imaging modalities.

A recent study [20] compared blood and urine specimens collected from patients with cancer-free control people using an AI model to examine droplet patterns. This study’s AI-assisted model uses a ResNet, which is a deep neural network (DNN) pre-trained on ImageNet datasets. The recognition and classification of complex patterns formed by dried urine or blood droplets under various circumstances led to a precise and sensitive cancer diagnosis. Using this technique consistently across droplets, comparisons may be made to uncover common spatial behaviors and underlying morphological features.

A hybrid methodology integrating statistical machine learning techniques with pre-trained DNN for feature extraction is proposed for BC classification tasks in [21]. This method may differentiate between BC tissue and normal tissue, muscle-invasive BC (MIBC), non-muscle-invasive BC (NMIBC), and post-treatment changes (PTCs).

In the medical field, the well-known U-Net neural network often complements its encoder with ResBlock from ResNet and Dense Block from DenseNet, as in [22]. This made the training possible while saving the parameters and lowering the overall recognition time. When used together with Attention Gates, the decoder is able to reduce irrelevant parts of the image and concentrate on important details. The authors presented a Residual-Dense Attention (RDA) U-Net model that was integrated with the earlier-mentioned model to delineate organs and spot lesions in CT-scanned abdominal images. The accuracy (ACC) for this model in the bladder and its lesions is thus 96% and 93%, respectively.

Di Sun et al. in [23] showed that the combination of radiomics and DL descriptions with clinical characteristics holds potential for improving the prognostication of 5-year survival rates in patients with BC post-radical cystectomy, but bigger datasets are required. Accurately estimating 5-year survival is advantageous when combined with surgical monitoring techniques and patient counseling.

By freezing specific DL-CNN layers and modifying the DL-CNN architecture, the authors of [24] examine the effectiveness of various DL-CNN models for BC therapy response evaluation based on transfer learning (TL). Therein, 123 chemotherapy patients’ pre- and post-treatment CT scans were gathered. Following treatment, 33% of patients had cancer in the T0 stage. Hybrid pre–post-image pairings (h-ROIs) were created by combining regions of interest from segmented lesions in pre- and post-treatment images. It was possible to collect test sets (54 pairs), validation (10 pairs), and training (94 pairs; h-ROIs, 6209).

Using full-slide digitized histologic images from two cohorts, Zheng et al. [25] developed a poorly supervised DL model for BC detection and overall survival prediction in muscle-invasive BC patients. Positive outcomes demonstrated that the proposed models can help physicians diagnose BC more accurately and make personalized treatment decisions for patients with muscle-invasive BC by facilitating differential risk classification. To extract more information from pathological images, further analysis can be performed on the most critical regions for diagnosis and prediction. Lastly, using the anticipated risk scores, the authors found six genes strongly associated with cancer advancement, which may lead to the identification of biomarkers.

MSEDTNet [26] is an auxiliary segmentation method introduced recently that merges a multi-scale encoder and decoder with a transformer. The multi-scale pyramidal convolution (MSPC) encoder is designed to create compact feature maps that could best retain the details of intricate local information within the image. The transformer constraint is used to model the interdependence of high-level tumor semantics in a global context over large distances. The decoder with a spatial context fusion module (SCFM) integrates context and refines high-resolution segmentation results. For T2-weighted MRI of 86 patients, MSEDTNet achieved an overall Jaccard index of 83.46% and a Dice similarity coefficient of 92.35% and demonstrated low complexity compared to the other models.

Using CNN, a method for detecting BC from CT images is provided in [27]. The frontal, horizontal, and sagittal plane image data sets are the three primary components of the image data set that are employed. CNN architectures that have already been described are used to categorize images. A five-fold cross-validation method is employed to assess CNN performances, providing insights into classification and generalization capabilities.

A new diagnostic method for BC that employs a Multi-Layer Perceptron (MLP) alongside a Laplacian edge detector is presented in [28]. This study evaluates the viability of using a more straightforward approach (MLP) in conjunction with prevalent techniques (DL-CNN) for BC identification. The research utilized a database comprising 986 photos of non-cancerous tissue and 1997 images of BC. The study’s results indicated that when trained and evaluated on pre-processed pictures using Laplacian edge detectors, MLPs can attain an AUC value of up to 0.99.

The investigation by Qaiser et al. [29] focuses on the practical use of AI models with region-level data lacking exhaustive patient annotation, solely for survival follow-up patient-level data. A weakly supervised survival CNN methodology (WSS-CNN) enhanced with a visual attention mechanism is developed to predict overall survival from whole-slide images of hematoxylin and eosin (H&E)-stained tumor tissue. The analysis of separated patient datasets for lung and bladder urothelial carcinoma indicated that WSS-CNN features predict overall survival for either type of cancer.

The study conducted by Mundhada et al. [30] classified urothelial carcinoma into low-grade and high-grade tumors according to the WHO 2016 classification. Transurethral resection of the bladder tumor (TURBT) specimens was digitally scanned in whole-slide images using H&E stain, low-grade, and high-grade non-invasive papillary urothelial carcinoma. These images were used as source images from which tumor tissue patches were extracted and subsequently used as input into a deep learning network. Patches that contained at least 90% of tumor tissue were used for modeling. For better accuracy regarding grading or classification of low- and high-grade urothelial cancer, hyper-parameter optimization of the deep learning model was carried out. Model performance was found robust after hyperparameter optimization with an overall accuracy of 90%. Grad-CAM and other visualization tools were also used to generate Class Activation Maps, indicating the proposition of employing this model as a companion diagnostic tool for grading urothelial cancer.

In 2020, a study proposed a machine learning system capable of differentiating between non-invasive Ta and superficially invasive T1 stages of BC [31]. This task poses significant challenges for pathologists due to the similarities in their microscopic appearance. The study involved the collection of 1177 images of bladder tumor tissues, comprising 460 non-invasive (Ta) and 717 invasive (T1) samples, and employed automated pipelines to extract features specific to T1 stage BC. Initial unsupervised clustering analysis did not successfully differentiate between Ta and T1 tumors; however, a refined set of features facilitated accurate classification at 91–96% using six supervised learning methods. Models utilizing manual feature extraction demonstrated superior performance to CNN models, indicating the significance of feature extraction informed by domain knowledge.

Lee et al. presented a preclinical (rat model) study that used surface-enhanced Raman spectroscopy (SERS) of urine samples with machine learning classification to detect early and polyp-stage BCs [32]. In the rat model, diagnostic accuracy was reported to be 99.6%. Hosney et al. introduced a hybrid diagnostic framework termed mRIME-SVM [33]. They used a Support Vector Machine (SVM) classifier for classification after improving the RIME metaheuristic method (by incorporating orthogonal learning) for feature selection. They used this technique to optimize SVM hyperparameters and choose discriminative features across several BC datasets. Based on their findings, mRIME-SVM performs better than other metaheuristic-based feature selection techniques and produces excellent classification accuracy in all datasets. To avoid the additional expense and delays associated with IHC or molecular testing, Jiao et al. built a DL framework to directly infer HER2 expression status from normal H&E-stained histopathology slides [34]. They employed a clustering-constrained attention multiple instance learning model on a sample of 106 BC cases (with known HER2 status). Their accuracy on the validation set was reported to be 0.86.

### Synthesis and Gap Identification

Table 1 compares the approaches discussed for identifying BC. Despite significant advancements in applying deep learning (DL) techniques to BC (BC) diagnosis, several critical gaps remain unaddressed in the current literature:

- Limited Use of Advanced Object Detection Algorithms: Most existing studies focus on classification tasks using CNNs and their variants for BC detection and grading. For instance, methods employing pre-trained CNNs for feature extraction [21], U-Net architectures for segmentation [22], and CNN-based classification from CT images [27] are prevalent. However, there is a lack of research utilizing advanced object detection algorithms like the YOLO family, specifically YOLOv11, for precise lesion detection and classification in histopathological images of BC.
- Insufficient Model Interpretability: While deep learning models have achieved high accuracy rates, they are often criticized for being “black boxes” with limited interpretability. Some studies have started to address this using visualization techniques like Grad-CAM [30] and attention mechanisms [29], but these efforts are not widespread. There is a need for integrating robust interpretability methods, such as EigenCAM, to provide visual explanations of the models’ decision-making processes, thereby enhancing trust and transparency in clinical applications.
- Underutilization of Histopathological Data: Several studies focus on imaging modalities like CT and MRI scans [22,23,24,26,27] or genomic data [31], with fewer using histopathological images for BC diagnosis. Histopathology remains the gold standard for cancer diagnosis, and utilizing this data can improve diagnostic precision. Studies that do use histopathological images often lack advanced techniques for both detection and interpretability.
- Need for Comprehensive Diagnostic Approaches: Current research often isolates different stages of the diagnostic pipeline, such as feature extraction [21,28], classification [27,30], or survival prediction [23,25,29], without integrating them into a cohesive framework. There is a gap in developing a holistic approach encompassing data collection, pre-processing, advanced modeling, interpretability, and performance assessment to streamline and enhance the diagnostic process.
- Limited Accuracy in Multi-Category Classification: Some studies achieve high accuracy in binary classification tasks [30,31], but there is a need for models that can accurately classify multiple categories relevant to BC diagnosis, such as distinguishing between inflammation, urothelial carcinoma (UCC), and invalid tissue samples.

The gaps thus identified will lay the foundation for the development of a complete, interpretable, and deep learning model for BC diagnosis, which shall be aimed at the following:

- Using YOLOv11 for Lesion Detection and Classification: The introduction of an object detection algorithm, YOLOv11, into the domain of BC histopathological image analysis for accurate lesion detection and multi-class classification.
- Enhancing Model Interpretability with EigenCAM: EigenCAM will be integrated to produce Class Activation Maps (CAMs), thus providing visual explanations for important pixels or regions that have affected the prediction made by the model. This will help lay bare the challenge of interpretability.
- Specialized Histopathology Data: The use of different histopathology slides stained in hematoxylin and eosin that include various stages and subtypes of BC to ensure that the model is exposed to apt and diverse data for training.
- Structuring an Interdisciplinary Diagnostic Scheme: An amalgamation of data collection, preprocessing, advanced modeling using YOLOv11, interpretability with EigenCAM, and exhaustive performance evaluation into a single framework to streamline the detection of BC with efficacy and accuracy.

The proposed work seeks to contribute substantially to the field of BC diagnosis by addressing these gaps, offering a non-invasive, rapid, and objective assessment tool that is both accurate and interpretable, ultimately improving clinical outcomes and patient care.

## 3. Methodology

**Figure 1:**
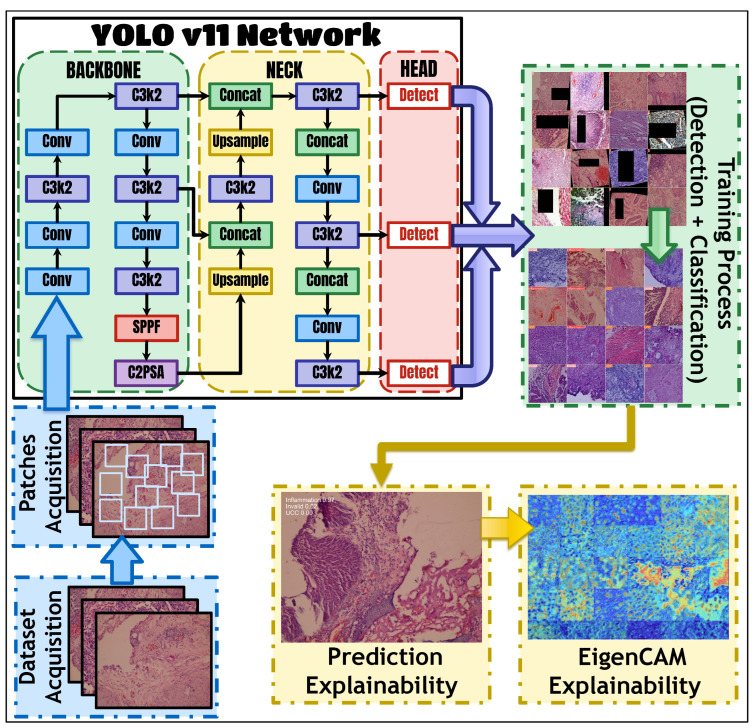
The proposed framework for BC diagnosis and interpretation using histopathology data.

The framework illustrated in Figure 1 provides a conceptual end-to-end pipeline for bladder cancer diagnostics using computational imaging. It outlines the key stages involved in a typical diagnostic workflow, including data acquisition and pre-processing, YOLOv11-based classification, interpretability using EigenCAM, and comprehensive performance evaluation. These components are described to demonstrate how the proposed system operates; however, no new images, samples, or clinical data were collected as part of this study.

As for data acquisition and pre-processing, the present study used a dataset with hematoxylin-and-eosin-stained histopathology slides of urinary bladder lesions. Several images were taken systematically from the slides to produce images that were categorized into inflammation, UCC, and invalid. Patients obtained these specimens from pathology departments, resulting in a dataset of 90 slides, out of which 43 showed cystitis and 47 UCC at different pathological stages. A digital camera mounted on a light microscope was used for imaging, and the images were manually classified into categories by a pathologist.

In fact, the classification method adopted was that of YOLOv11 for bladder prediction, which was meant to classify the histopathology slide to histopathological categories of inflammation, UCC, or invalid. The method YOLOv11 is one of the best in the object detection domain. It incorporated some architectural improvements including anchor-free detection, new convolutions, and mosaic augmentation. Anchor-free detection does away with predefined anchor boxes, while new convolutions optimize feature extraction. Mosaic augmentation enhances model robustness when exposed to a variety of contexts. The Ultralytics YOLOv11 package was used in this study, incorporating pre-trained classification models into it.

Interpretability using EigenCAM provides insights into classification tasks by generating Class Activation Maps (CAMs) to discern influential pixels or regions in images. EigenCAM facilitates efficient interpretation without requiring model modifications, integrating seamlessly with CNN models. It employs Singular Value Decomposition (SVD) to derive Class Activation Maps, enabling visualization of significant image regions contributing to classification decisions.

Performance analysis in BC classification evaluations incorporated different metrics like accuracy, precision, recall, specificity, F1 measure, entire intersection over union (IoU), balanced accuracy (BAC), Matthews correlation coefficient (MCC), Youden’s J statistic, and Yule’s Q statistic. These metrics give a full view on model effectiveness concerning accuracy, sensitivity, specificity with performance on imbalanced datasets.

### 3.1. Data Acquisition and Pre-Processing

The dataset used in this study is titled “Digital Histopathology Image Dataset of Hematoxylin- and Eosin-Stained Bladder Urothelial Cell Carcinoma versus Inflammation”. The dataset is publicly available and fully anonymized and can be accessed at https://doi.org/10.5061/dryad.0cfxpnw5q. In this work, we performed secondary analysis only on these de-identified histopathology images. No new human data, biological specimens, or patient information were collected or accessed during this research.

While comprising 90 whole-slide images (WSIs), the dataset provides 14,891 non-overlapping patches, which constitute a substantial number of training samples for deep learning models. As to patient selection, the specimens were from the Departments of Pathology from both the Faculty of Medicine and Cancer Institute at Assiut University. Thus, the dataset holds 90 hematoxylin and eosin histopathology slides, sectioned and preserved with formalin and with cystitis diagnosed in 43 slides and UCC in another 47 slides. The slides also represented UCC patients in various pathological stages: pTa (5 slides), pT1 (9 slides), pT2 (28 slides), and pT3a (5 slides). The Olympus E-330 digital camera was used to photograph the slides, mounted on a light microscope-CX31- by Olympus, with the Olympus E330-ADU1.2X adapter. The magnification of the microscope was set to 20×, and camera settings were adjusted prior to photography. The resolution of the images was 3136 × 2352 pixels, it was in JPEG format, and it had a 1:2.7 compression rate. To mitigate potential overfitting inherent in smaller datasets, we employed extensive data augmentation strategies, including flipping, rotation, and mosaic augmentation, as recommended by Ameen et al. [35] for digital histopathology tasks. These techniques have been shown to effectively enhance model generalization. Furthermore, the balanced distribution across classes (5948 inflammation, 5814 UCC, and 3131 invalid) helps ensure robust performance.

All images in the dataset were originally annotated by an expert pathologist into three diagnostic categories: inflammation, UCC, and invalid. Inflammation images were diagnosed as having an inflammatory cell infiltrate, UCC images were based on malignant urothelial cells, and invalid images represent tissue samples that do not meet diagnostic criteria for either inflammation or UCC. This category encompasses specimens with significant artifacts (e.g., folding, staining irregularities, excessive debris), areas of poor tissue quality (e.g., necrosis and autolysis), or regions lacking sufficient cellular detail for reliable classification. Critically, this class holds significant clinical relevance, as pathologists routinely encounter such suboptimal samples in daily practice. Distinguishing “invalid” tissue from true pathology prevents false-positive diagnoses and improves the overall reliability of the diagnostic system.

**Figure 2:**
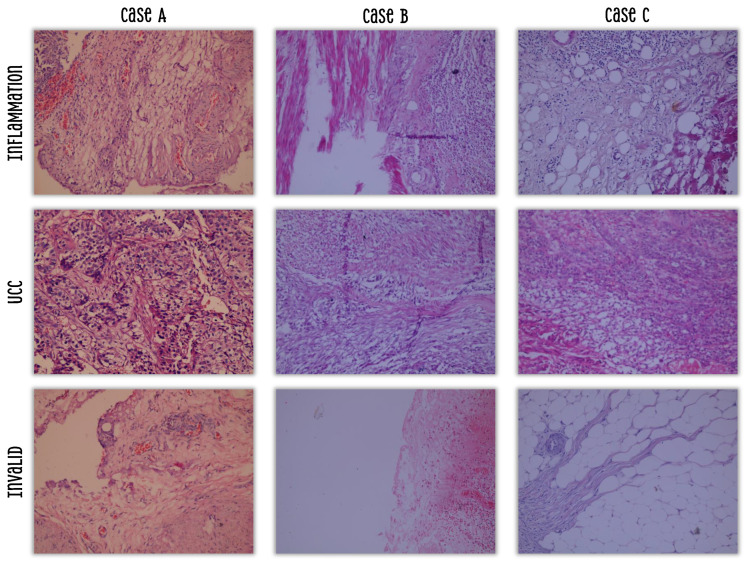
Samples from the used dataset.

Sample images are presented in Figure 2 [36]. Moreover, to prevent data leakage, all patches derived from a single WSI were assigned to the same subset (training, validation, or test). The 90 WSIs were randomly partitioned into training (70%), validation (15%), and test (15%) sets. This slide-level splitting ensures that no information from the test set can influence the training process, guaranteeing a fair evaluation of model generalization.

### 3.2. YOLOv11 Classification for Bladder Prediction

While YOLOv11 was originally designed for object detection, its architectural innovations make it exceptionally well-suited for high-fidelity classification tasks in medical imaging, particularly when interpretability and computational efficiency are paramount. Unlike traditional classification backbones (e.g., ResNet and EfficientNet), YOLOv11 incorporates unique components like the C3k2 block and C2PSA (Convolutional block with Parallel Spatial Attention) spatial attention module, which are specifically designed to enhance feature extraction and focus on diagnostically relevant regions [37]. The C3k2 block reduces computational overhead while maintaining feature richness, making it ideal for resource-constrained clinical settings. The C2PSA module dynamically highlights critical spatial features, providing an intrinsic mechanism for interpretability that aligns perfectly with our goal of creating a transparent diagnostic tool. Furthermore, using YOLOv11 allows for a seamless transition to full object detection if future work requires precise lesion localization, offering a more versatile foundation than static classification networks. Therefore, our choice is not merely to use a classifier but to utilize a framework inherently designed for both precision and explainability in complex visual domains.

The improvements to the architecture of YOLOv11 are the ones that lead to the increased performance of the model. The C3k2 block is a main component, and it is the one that changes the C2f block that was used in previous versions like YOLOv8. The C3k2 block is a less expensive version of the Cross-Stage Partial (CSP) bottleneck in terms of computations. Instead of one big convolution, it uses two small convolutions, and, thus, the computational overhead is significantly reduced, while the ability to extract features is kept at the same level or is even improved. Such a performance is very important for applications in medicine where it might be necessary to carry out very fast inference on a system that has limited resources. Moreover, through the addition of the C2PSA component, an impressive spatial attention method was introduced to the model. In the C2PSA module, the spatially pooled features are what the model looks at most intensively, paying attention to the most important image regions [37]. These features are, without a doubt, very useful in medical imaging, where the smallest pathological features can make a huge difference and can be localized to some specific areas only. With the C2PSA module, pathologists obtain the power to pinpoint these regions and hence drastically improve the chances of finding the pathologies that are hard to detect or are partially hidden and can be used in diagnosing the bladder problem.

The YOLOv11 architecture follows the standard YOLO pattern, comprising three primary components: (1) the backbone, responsible for extracting low- and mid-level features from the input image; (2) the neck, which aggregates multi-scale features from the backbone for enhanced localization; and (3) the head, which produces the final predictions (class scores and bounding boxes). In our adaptation for classification, we employ the entire architecture, utilizing the backbone and neck for powerful feature extraction and the head for classification. The C3k2 blocks within the neck and head are optimized for efficient multi-scale feature processing, while the C2PSA module in the neck enhances spatial attention.

In the neck and head parts, YOLOv11 has several C3k2 blocks to perform the multi-scale feature maps processing and refinement in a very efficient way. The C3k2 block, depending on how it is configured, can either function as a normal bottleneck or a deeper C3 module and hence provide the user with the option of deciding the extent of the feature extraction. Moreover, to additionally stabilize data flow and feature extraction, the head section after the C3k2 blocks also has CBS (Convolution–BatchNorm–SiLU) layers, and for feature extraction, the SiLU activation function is used, which, as experiments have shown, leads to improved performance of the model.

The ability of the model to find the correct predictions even in very difficult cases and the fact that it has been successful in a variety of computer vision areas make it a very interesting option for the creation of powerful and, at the same time, efficient instruments for the automatic bladder condition evaluation. One of the main points of the refined architecture of YOLOv11 that makes it so powerful is the capability of the system to extract features if need be, even those that are hidden deeply in some complex images. As an example, YOLOv11m is able to deliver better mean Average Precision (mAP) results over standard benchmark datasets while also being able to perform with 22% less YOLOv8m parameters, thus strengthening the computational efficiency side, and still, no accuracy is given up. Such a parameter efficiency is indispensable when thinking of model deployments in clinics where the computational power may not always be enough.

By employing C2PSA, the model is further improved in terms of how it can adjust to the context and understand it, whereby it can better analyze and interpret complex visual information. Medical image classification is a perfect example to which it can be applied, wherein the model is required to detect highly detailed patterns that are the diagnostic indication of the disease. The deployment flexibility of the model capabilities at different scales, for example, from the lightweight nano variant to the high-accuracy xlarge variant, is one very big advantage that the model opens up, for instance, in clinic environments that are different from each other and where the performance has to be at the same level [37].

### 3.3. Interpretability with Eigen-CAM

EigenCAM generates Class Activation Maps (CAMs) by performing Singular Value Decomposition (SVD) on the activation maps of the final convolutional layer. SVD decomposes the activation matrix into orthogonal components, with the first singular vector representing the direction of maximum variance. By projecting the activation maps onto this principal component, EigenCAM identifies the most influential spatial regions contributing to the prediction. This method is advantageous because it does not require gradients, making it computationally efficient and universally applicable to any CNN architecture without modification. Its output is a heatmap that highlights the pixels or regions most salient to the model’s decision, providing a transparent view into the model’s reasoning process.

Eigen-CAM in comparison has an intuitiveness to it and is indeed user-friendly to apply, computationally efficient to compute, and unconstrained by correct model classification details. This method operates with nearly all the CNN models without bringing any alterations to it concerning layers or conducting retraining. Eigen-CAM becomes even more advanced among all types when constructing visual explanations for predictions performed by the CNN. It ensures consistency and class separability above most recent works and, in addition, being tolerant of some properly predicted erroneous classifications, which often stem from the dense layer [38].

Eigen-CAM highlights the pixel analysis of object detection. In the heart of the feature extraction network are the convolutional layers that begin with explicitly lower spatial features such as edge and angle detection. As it goes up the hierarchy, it becomes more enigmatic, feeding exclusively on high-level general features that carry semantic meaning, with low-level item-based meanings that are hard to extract. In displacement, on the other hand, when convolution networks are fed into the fully connected layers, emotionally learned features are extended, and the model is given stepping boundaries for separating notions [39].

Consider an input image *I* of size i×j, $I\in R^{i,j}$. Here, $W_{L}=n$ denotes the concatenated weight matrix of the first *k* layers of size (m,n). The class-activated output $O_{L}$ was obtained by projecting the image *I* onto the last convolution layer L=k, such that $O_{L}=K=W_{L}^{T}\times I$. Singular Value Decomposition (SVD) was used to factorize $O_{L}=K$ into its main components, with the factorization being written as $O_{L}=K=U\times \Sigma V^{T}$, where *U* is an orthogonal M×M matrix, its first column being left a singular vector. Σ is a diagonal M×N matrix, having singular values on its diagonal, and *V* is an orthogonal N×N matrix, its first column again being a singular vector. The Class Activation Map $L_{EigenCam}$, is obtained by projecting $O_{L}=K$ onto the first eigenvector $V_{1}$ such that $L_{EigenCam}=O_{L}=K\times V_{1}$ [40,41].

### 3.4. Performance Evaluation

We applied a variety of performance metrics to evaluate the effectiveness of models in classifying BC with YOLOv11. The performance parameters are given here, which describe one or more facets of classification performance, namely accuracy, precision, recall, specificity, F1 score, intersection over union (IoU), balanced accuracy (BAC), Matthews correlation coefficient (MCC), Youden’s J statistic, and Yule’s Q statistic.

Accuracy measures how well the correctly classified instances compare with the total number of instances in the dataset. Therefore, it provides a holistic view of how well a model performs but may fail completely when dealing with an imbalanced dataset.

Precision is a ratio of true-positive predictions to all positive predictions. It suggests how well the model can avoid false-positive predictions. Recall or sensitivity refers to the ratio of true-positive predictions to the total count of actual positives. Such metrics tell how well the model can successfully achieve all positive instances from within the dataset. Specificity refers to the number of negative predictions correctly identified to the corresponding total number of actual negatives. This performance measure emphasizes the model’s prediction accuracy in finding negative instances.

F1 score represents the harmonic mean of precision and recall; effectively, this metric balances precision and recall to assess the model’s performance in totality. intersection over union (IoU) measures the degree of overlap between the predicted bounding boxes and the bounding boxes defined in the ground truth. That is the reason accurate localization counts in object detections such as BC classification.

For imbalanced datasets, BAC takes away the average gain of sensitivity (recall) and specificity. This provides a more reliable assessment of classification performance when there is disproportionate class distribution. MCC evaluates classification performance by drawing on true positives, false positives, and false negatives. Thus, it is particularly useful for imbalanced datasets, allowing an even appraisal of the model’s predictive ability for each class.

Youden’s J statistic combines sensitivity and specificity into one metric to evaluate classification performance. It ranges from 0 to 1 with higher values indicating better performance. Yule’s Q statistic quantifies the relationship between predicted and actual classifications. This can be applied in assessing the strength and direction of predictive relationships.

## 4. Experiments and Discussion

Experiments were conducted in a Windows 11 (64-bit) OS environment with a NVIDIA GeForce RTX 3050 (4 GB VRAM) manufactured by NVIDIA Corporation (Santa Clara, CA, USA), and 128 GB RAM. Although relatively modest, this setup was sufficient for training and evaluating the YOLOv11 models on image patches rather than whole-slide images. The software environment included Python 3.10, the Ultralytics YOLOv11 framework for model development, and PyTorch (version 2.1.0) with CUDA-enabled acceleration. EigenCAM, implemented through PyTorch-CAM, was used to generate Class Activation Maps and provide visual interpretability of model predictions. Additional libraries such as OpenCV, NumPy, SciPy, Pandas, and scikit-learn supported preprocessing, numerical computation, and performance evaluation. All development and experimentation were conducted using Jupyter Notebook (version 7.0.6) and Visual Studio Code (version 1.85.1) on Windows 11. For model evaluation, we used five variants of the YOLOv11 architecture, YOLOv11-nano, YOLOv11-small, YOLOv11-medium, YOLOv11-large, and YOLOv11-xlarge, which differ in their parameter counts.

Ameen et al. [35] introduced a publicly available dataset of non-overlapping urinary bladder histopathology slides categorized as inflammation, UCC, or invalid (see Section 3.1). To address the scarcity of annotated data, they explored 11 augmentation strategies, including flipping and rotation, and evaluated four CNNs on binary classification tasks using accuracy, sensitivity, specificity, and AUC. Their findings highlighted that the most effective strategy was to apply augmentation after separating the test set but before splitting training and validation data, with Inception-v3 achieving the highest accuracy. The study emphasized the critical role of augmentation in digital histopathology and suggested further validation for broader applicability [35]. Unlike Ameen et al. [35], who excluded the invalid category, our study utilizes all three classes (inflammation, UCC, and invalid).

In Table 2, we have detailed a comparison of five classification models of YOLOv11 over seven metrics including precision, recall, F1 score, accuracy, specificity, balanced accuracy (BAC), and an average score. The outcomes depict a progressive trend of performance improvement as the model grows from nano to large, and then levels off for the extra-large variant. The YOLOv11-large (YOLO11l) model is, therefore, the best selection for this particular job as it attains the maximum scores not only in each individual metric but also in the overall average. To assess the robustness of these results, we performed 5-fold cross-validation on the entire dataset. The mean accuracy across the five folds was 96.8 ± 0.4%, with a 95% confidence interval of [96.0%,97.6%]. Similarly, precision and recall showed high consistency, with means of 95.2 ± 0.3% and 95.3 ± 0.3% respectively. These narrow confidence intervals indicate that the model’s performance is stable and reproducible, mitigating concerns about overfitting to a single train/test split.

Those results go hand in hand with the YOLOv11 design principle whereby bigger models like the “large” and “xlarge” versions are intended to discover more complex features by means of deeper networks and higher parameter counts, thus increasing their ability of subtle pattern recognition in medical images. Furthermore, the excellence of YOLO11l is due to its redesigned backbone and neck, which are more efficient and also more advanced structures like the C3k2 block and C2PSA module. The C3k2 block’s rapid feature extraction and C2PSA spatial attention mechanism allow the model to zero in on those parts of the histopathology slides that are of diagnostic value, which results in highly accurate predictions. Besides that, the model’s near-identical precision and recall (both 95.47%) suggest that it is very well-balanced with very little bias towards a particular class, which is a very important feature in clinical decision support systems.

**Figure 3:**
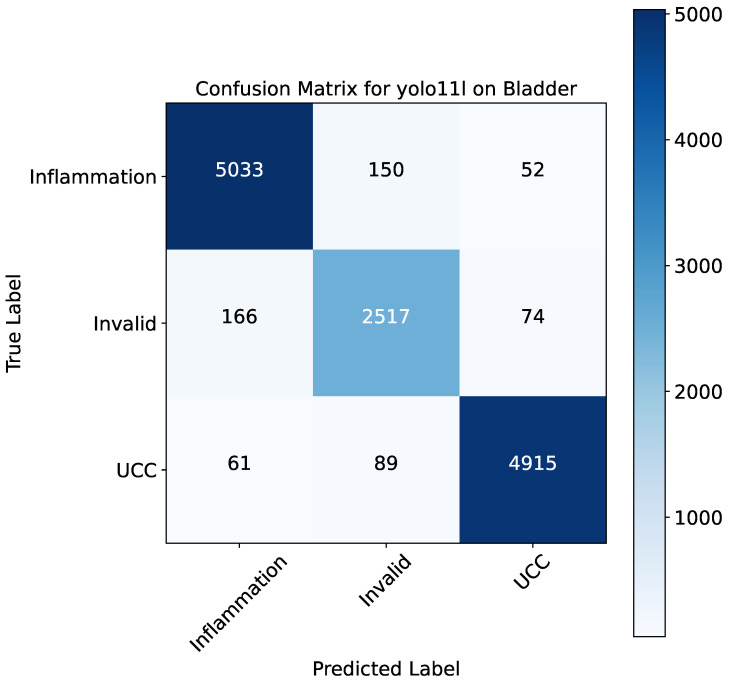
Confusion matrix for the YOLOv11-large model on the bladder histopathology classification task. The matrix details the number of true positives (diagonal) and misclassifications (off-diagonal) for the three classes: inflammation, invalid, and UCC.

**Figure 4:**
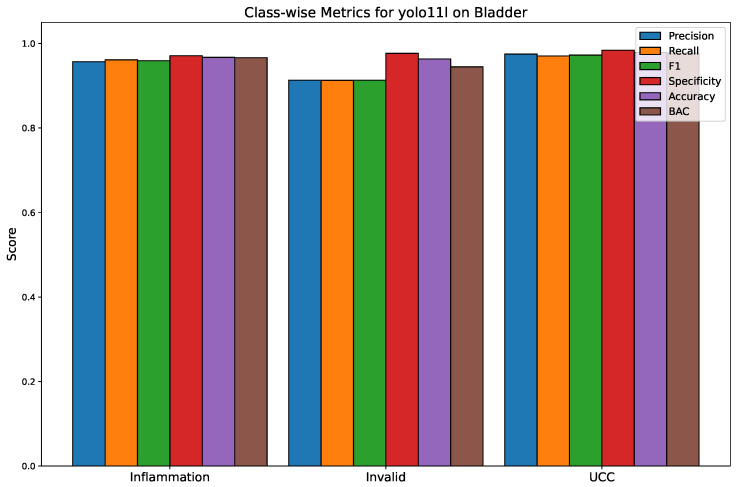
Class-wise performance metrics for the YOLOv11-large model. This bar chart compares the precision, recall, F1 score, specificity, accuracy, and BAC for each of the three classes: inflammation, invalid, and UCC.

Figure 3 depicts the confusion matrix of YOLO11l, which is a detailed snapshot of its classification performance. The matrix points to the high true positive rates for the three classes, with most of the predictions being on the diagonal. To be more precise, the model finds 5033 of 5235 inflammation (TPR = 96.13%), 2517 of 2657 invalid (TPR = 94.67%), and 4915 of 5065 UCC (TPR = 97.01%) correctly. The off-diagonal elements of the matrix suggest that these particular positions are the main sources of misclassification: as a result, a small number of inflammation cases were misclassified as invalid (150) or UCC (52), while in the other direction, a similar trend can be seen for the classes. This means that on the one hand, the model is great at separating healthy from diseased tissue, but on the other hand, there is still some uncertainty about the difference between inflammation and invalid due to shared morphological features. Moreover, Figure 4 shows the class-wise performance for each class and for each model.

**Figure 5:**
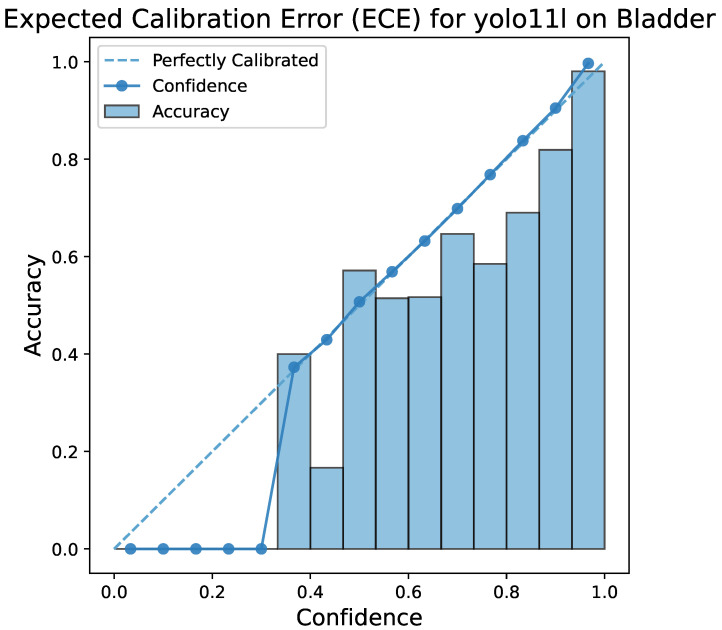
ECE plot for the YOLOv11-large model. This figure visualizes the relationship between the model’s predicted confidence and its actual accuracy, demonstrating that the model is well-calibrated for all three classes.

Figure 5 demonstrates the Expected Calibration Error (ECE) of the YOLO11l model. A small ECE figure means that the model’s predicted probabilities are in good agreement with the actual outcomes; e.g., if the model predicts the class with 95% confidence, it is correct 95% of the time. Such calibration is very important for the acceptance of a decision support system in medical diagnosis because clinicians not only depend on the predicted label but also on the confidence score for making their decisions.

**Figure 6:**
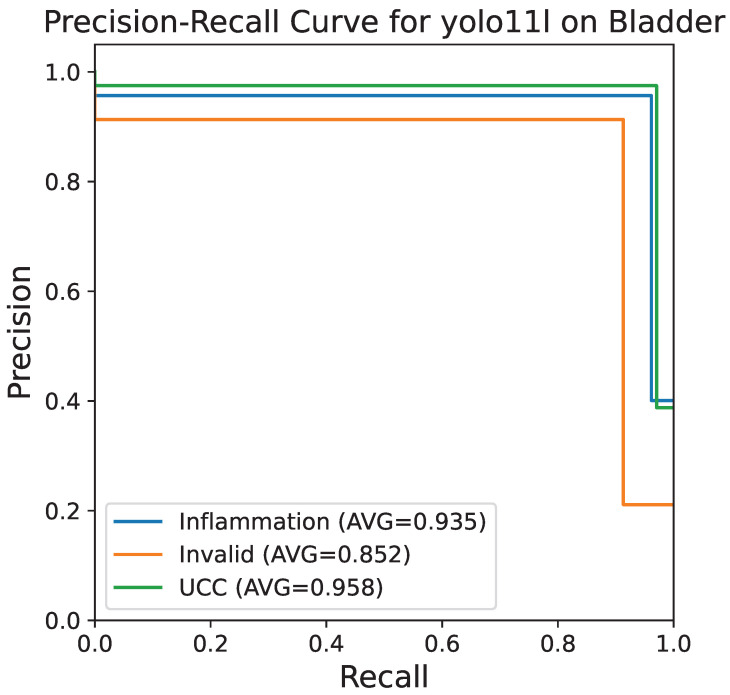
PR curve for the YOLOv11-large model on the bladder histopathology classification task. The area under the PR curve for each class is shown: inflammation (0.935), invalid (0.852), and UCC (0.958), indicating high performance even on imbalanced data.

Figure 6 shows the precision–recall curve, which is very useful in the case of imbalanced datasets. The class-specific AUC scores are very close to the maximum value with inflammation being 0.935, invalid 0.852, and UCC 0.958 on average. These excellent AUC values are evidence that the model is extremely strong in keeping high precision even at high recall levels, which is necessary for a diagnostic context where false negatives need to be minimized.

**Figure 7:**
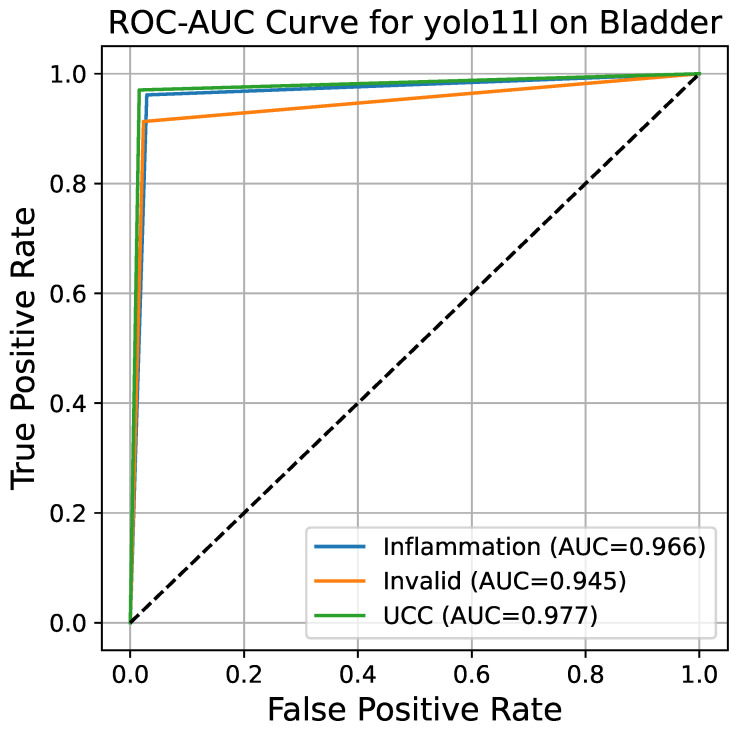
ROC curve for the YOLOv11-large model. The overall AUC score of 0.972, calculated using the One-vs-Rest (OvR) method, confirms the model’s outstanding ability to discriminate between the three classes of bladder histopathology.

Figure 7 represents the ROC curve together with the AUC for the YOLO11l model. The ROC curve shows the true positive rate (TPR, or sensitivity) as a function of the false positive rate (FPR, or 1 − specificity) at different classification thresholds. The overall AUC for the multi-class problem is derived by the One-vs.-Rest (OvR) method, resulting in a value of 0.972. Such a high AUC score indicates that the model possesses an excellent capability of differentiating between the three classes; therefore, its performance is far better than that of a random guess (which would result in an AUC of 0.5). The almost perfect curves for the inflammation and UCC classes, together with a slightly lower but still very strong curve for the invalid class, confirm the model’s general power of diagnosis.

**Figure 8:**
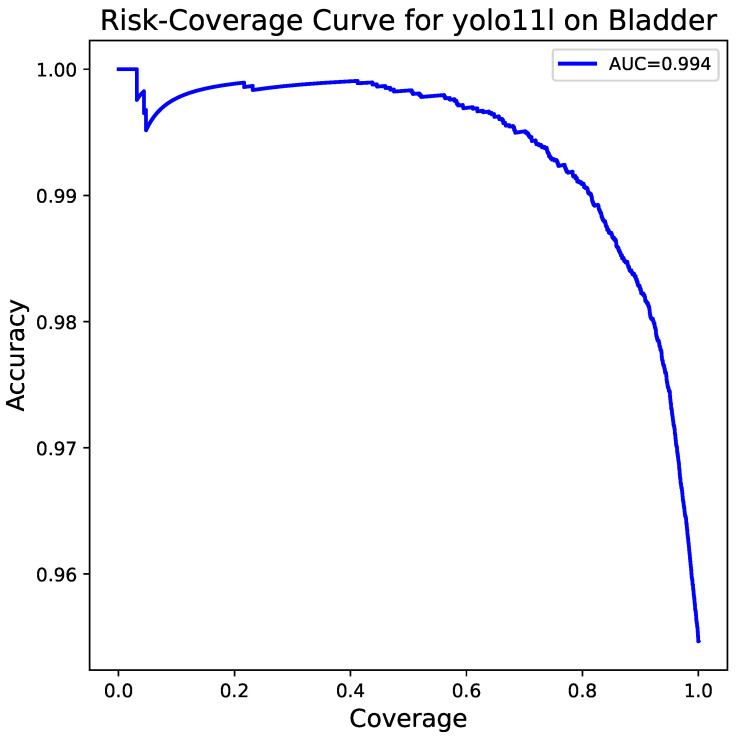
Risk–coverage curve for the YOLOv11-large model. The AUC of 0.994 demonstrates the model’s exceptional reliability, maintaining high accuracy across a wide range of coverage thresholds.

At last, Figure 8 shows the risk–coverage curve which measures how well a model performs as the risk it takes increases. The curve depicts a very small (0.006) area under the curve (AUC) or, conversely, an area of 0.994 demonstrating that the model stays very accurate even if the covered portion of the data is large. Thus, it not only points out the model’s dependability but also its potential use in scenarios of the real world of clinical practice where a high degree of certainty is the requirement.

That said, our experiments lend support to the view that the YOLOv11-large model can be employed successfully for the multi-class classification of bladder histopathology slides. It outperforms the previous methods and makes use of the cutting-edge architectural elements of the YOLOv11 framework to yield precise, dependable, and well-calibrated predictions.

**Figure 9:**
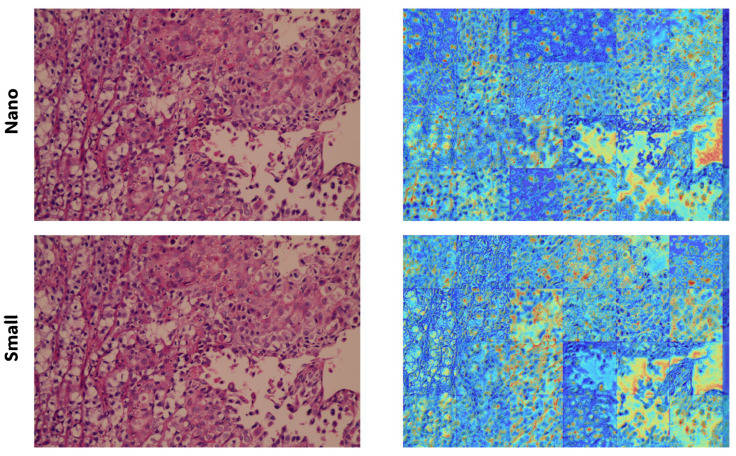
The EigenCAM interpretation on a testing histopathology sample using the YOLOv11.

Additionally, Figure 9 demonstrates how EigenCAM interprets a testing sample. EigenCAM operates on each tile of the large histopathology image, offering graphical inference. This interpretation is conducted on the final 13 layers of the YOLOv11 architecture, enhancing the descriptive quality of the interpretation.

### 4.1. Comparison with Established Classification Architectures

To establish the relative advantage of the proposed YOLOv11 framework, we conducted a comparative analysis against several established deep learning classification architectures: ResNet-50, DenseNet-121, EfficientNet-B4, and Vision Transformer (ViT-Base). All models were trained and evaluated on the identical dataset and experimental setup (same train/val/test split, same augmentations, same hardware). The results are presented in Table 3. Based on this analysis, the YOLOv11-large model outperforms all baseline architectures across all key metrics. Its superior performance can be attributed to its specialized architectural features (C3k2 and C2PSA), which enhance feature extraction and spatial attention, leading to better discrimination of subtle pathological patterns compared to generic classification backbones.

### 4.2. Medical Importance of the Study

This study fulfills an important unmet requirement in urologic oncology: the ability to efficiently yet accurately diagnose bladder cancer objectively based on histopathology slides. BC is one of the top 10 global malignancies with high rates of recurrence and significant mortality when not diagnosed early. Current diagnostic procedures that necessitate invasive interventions—such as cystoscopy and subjective interpretation by pathologists using H&E-stained tissue sections—are time-consuming, resource intensive, and inconsistent due to differences from one observer to another.

The proposed design is the YOLOv11-EigenCAM, which encompasses automated interpretation and highly accurate diagnostic tools. The model achieves an accuracy of between 93.81 and 96.49% and reliably classifies tissue in three clinical relevant categories: inflammation, UCC, and invalid/low-quality samples. Above all, this description reduces turnaround times in diagnostic procedures. Furthermore, it is clinically impactful, especially with triaging of cases, hence allowing pathologists to concentrate more on high-risk or questionable samples.

Such AI-enabled applications would seamlessly integrate into digital pathology systems at the actual clinical site for pre-screening. Fast assessment of malignant lesions could speed up intervention, which is one of the many ways to improve patient survival. A case in point would be such a network in high-volume pathology hubs or telepathology networks for rural and underrepresented areas, to prioritize urgent cases and clear diagnostic backlog while providing equally consistent assessments even where expert pathologists are few.

The addition of EigenCAM visualizes the model’s prediction with respect to specific tissue areas responsible for its decision. Such interpretability promotes trust amongst clinicians and backs the educational use of the medium for trainees and thus enables quality control that is possible through human–AI collaboration. This transparency guarantees that AI serves as a trusted co-pilot rather than a substitute, in accordance with regulations and ethical governance for clinical deployment of AI.

Besides primary diagnosis, very robust performance metrics—specificity (>94%), Youden’s J (>0.88), Yule’s Q (>0.98)—also delineate more applications such as treatment monitoring, recurrence prediction, and risk stratification under which the system may be used. It could evaluate therapeutic response or detect residual disease through serial biopsies or surgical specimens.

This work addresses the gap between cutting-edge deep learning and clinical practice. The introduction of widely available H&E-stained slides into personalized medicine with an interpretative output will see the advancement of the YOLOv11-EigenCAM framework in BC diagnostics as an innovation that is scalable, cost-effective, and trustworthy, thereby promoting better patient outcomes with less stress in the system.

### 4.3. Slide-Level Aggregation and Clinical Relevance

To bridge the gap between patch-level performance and clinical workflow, we implemented a simple slide-level aggregation strategy. For each WSI in the test set, predictions from all constituent patches were combined using majority voting. The final WSI diagnosis was assigned based on the most frequent predicted class among its patches. While slightly lower (see Table 4) than the patch-level performance, the slide-level accuracy of 94.44% demonstrates that the model retains strong diagnostic capability at the clinically relevant scale. This approach provides a practical output for pathologists, who typically evaluate entire slides rather than individual patches. Future work can explore more sophisticated aggregation methods (e.g., weighted averaging based on patch confidence) to further improve slide-level accuracy.

### 4.4. Complexity Analysis and Real-Time Implementation

The focus for the development of diagnostic accuracy and eventually clinical usefulness among high computational and practical implementations was also kept in mind in the architecture of the proposed YOLOv11-EigenCAM framework. Its complexity is mainly dependent on the architecture of the YOLOv11 models (in particular, the nano and small types) that were chosen in view of balancing the performance with the resource constraints.

YOLOv11-nano provides a lightweight solution in approximately 2.7 million parameters for gratifications such as the extreme limitation of edge devices or environments with minimal GPU memory, i.e., 4 GB NVIDIA GPU used in this study. The anchor-free detection and optimized convolutional-based modules (C2f and C3) diminish computational restraints while still performing solid feature extraction. In contrast, although YOLOv11-small (with 6.4 million parameters) provides extra accuracy at the price of higher computational requirements, it is more suitable for being deployed on server bases with fewer constraints toward processing power and memory.

Both architectures used mosaic augmentation to improve generalization during training without affecting inference time significantly. During inference, YOLOv11 works in real time or near real time with standard hardware, processing histopathology tiles rapidly enough to meet clinical workflow requirements. Since this study does not report exact FPS values owing to the static nature of whole-slide image analysis, it is nevertheless worth mentioning that the design of the model naturally supports rapid batch processing of tiled images, which is a critical requirement in the scanning of huge digital slides.

EigenCAM’s incorporation is granted during inference with minimal added computational effort. EigenCAM avoids backpropagation through dense layers like other gradient-based techniques such as Grad-CAM, but rather finds using SVD applied to activation maps of the last convolutional layer. It thus requires less computation and no retraining or architectural change of the model, and it runs in parallel with the classification output. As shown in Figure 9, EigenCAM provides insightful heatmaps within seconds for each tile, alleviating the burden on the clinicians, who, alongside the prediction, can quickly review visual explanations without jeopardizing diagnostic throughput.

For the real-time implementation in pathology labs, the system could be established either as a cloud-based or a local server application linked with digital slide scanners. Histopathology images are preprocessed into non-overlapping tiles (as in this study) that are then classified by YOLOv11. Results can be visualized with EigenCAM overlays and subsequently aggregated to produce slide-level reports with areas of inflammation, carcinoma, or invalid tissue for the pathologists to concentrate on suspicious regions. This paradigmatic human-in-the-loop boosts diagnostic security and alleviates workloads.

Future optimization by quantization, pruning, or tensorRT formats for faster inference on NVIDIA hardware is possible due to the modular design of the framework. The compatibility of open-source tools such as Ultralytics YOLOv11 and the PyTorch CAM library allows for scalability across institutions and adaptation to new datasets or cancer subtypes.

## 5. Conclusions and Future Directions

This study demonstrates the strong potential of modern deep learning architectures for multiclass bladder cancer histopathology, with the large model achieving state-of-the-art accuracy and excellent robustness across inflammation, UCC, and invalid categories. Its architectural innovations (efficient feature extraction and spatial attention) enabled precise and well-calibrated predictions, supported by high AUPRC and ROC–AUC scores. Although minor ambiguities emerged between inflammation and invalid samples, the overall discriminative performance was clinically compelling. Future work will focus on expanding dataset diversity to strengthen generalizability further, integrating multimodal clinical and molecular data, and deploying the framework within real digital pathology workflows enhanced by EigenCAM-based visual explanations. We also plan to explore model compression and optimization strategies to facilitate deployment in resource-constrained environments. Importantly, clinical validation by expert pathologists is planned as a key component of our forthcoming evaluations, ensuring that the system’s predictions align with real-world diagnostic expectations.

## Figures and Tables

**Table 1:** Comparison between different techniques for identifying BC.

Ref	Technique	Dataset	Performance	Pros	Cons
[20]	DL analysis of dried blood/urine droplet patterns.	Patient blood and urine droplet images for 110 male and 20 female	ACC of 0.973 for whole blood	Improved the accuracy and efficiency of diagnosing BC.	sensitive to pre-analytics (drying conditions, slide substrate)
[21]	Hybrid DL + classical ML	T2-weighted MRI images from 65 individuals	ACC of 78.50%, AUC of 80.60%	Use strengths of both ML, DL.	Dependent on annotated histology data
[22]	Residual-Dense-Attention U-Net	CT scans from 41 patients.	ACC of 96%	includes attention to suppress irrelevant areas.|Fast inference reported.	Small dataset, risk of overfitting.|Evaluation on more diverse CT scanners/hospitals needed.
[23]	Survival prediction combining clinical variables, radiomics features, and DL image descriptors (hybrid model).	CT urography images, clinical data, and histological information on 163 individuals.	AUCs of 0.82, 0.73, and 0.71 for the clinical, radiomics, and DL descriptors.	Support clinicians in making informed decisions about treatment strategies and patient care.|Can uncover hidden information and improve the predictive performance of the model.	Complexity of combining modalities; needs harmonization of imaging and clinical data.|Need to be generalized on large and diverse datasets to ensure robust predictions.
[24]	Assess treatment response (pre/post-chemo CT pairs) via TL with different DL-CNN variants.	CT scans from 123 patients (pre- and post-treatment pairs).	AUCs were 0.81, 0.78, and 0.71	Automated and efficient analysis of treatment response.|Lowering inter-observer variability and producing evaluations that are more dependable and consistent.	Limited sample size.|Imaging heterogeneity (timing, protocols) affects generalizability
[25]	Weakly-supervised deep-learning models on whole-slide H&E images for diagnosis and overall survival prediction (WSI-based DL).	TCGA includes 926 H&E-stained WSIs from 412 individuals with BLCA.	ACC of 0.987	Reducing the workload of pathologists and enabling more timely diagnoses and survival predictions.|High detection accuracy	Weak supervision can limit localization precision.|Performance depends on cohort composition (TCGA biases).
[27]	Using CNN for detecting BC from CT images.	Three image categories from lower abdominal CT scans with 4413 (frontal), 4993 (horizontal), 996 (Sagittal)	ACC of 0.98 frontal, 0.98 horizontal, 0.82 sagittal	Uses data augmentation to mitigate small datasets.|Improved diagnostic accuracy	Pre-processing choices strongly affect results.
[28]	MLP with Laplacian edge detector preprocessing.	986 images of non-cancer tissue and 1997 images of BC	AUC of 0.99	Integrating edge detection with an MLP improves the model’s classification ability.|Diagnose BC with high performance	High reported AUC may depend heavily on preprocessing and dataset composition.|Less robust to complex variability than deep models.
[30]	DL classification of urothelial carcinoma grades with visualization (CAMs) to aid interpretability.	Samples from 20 patients at SRMC, Chennai, who had NMIBC.	ACC of 90%	Improved diagnostic accuracy.|Enhanced visualization	Grade labels can be subjective and noisy.|Need to be generalized on large and diverse datasets to ensure robust predictions
[31]	Classical ML pipelines: to distinguish non-invasive (Ta) vs invasive (T1) BC.	1177 images of bladder tumor tissues, 460 non-invasive (Ta) and 717 invasive (T1).	ACC of 91–96%.	Handcrafted features capture meaningful histologic patterns.	Handcrafted features may miss complex morphological cues that deep models capture
[32]	SERS of urine on Au-coated chips + PCA + ML.	Preclinical rat model: urine from carcinogen-induced bladder tumors vs controls.	ACC >99.6%, AUC >0.996	Very high discrimination in controlled preclinical setting.|SERS provides molecularly rich signals.	Rat model results may not translate directly to humans.|SERS signals are sensitive to substrate and sample prep
[33]	Improved RIME metaheuristic with Orthogonal Learning for feature selection + SVM classifier.	9 BC datasets from official repositories	Minimum average accuracy >92%	Focus on feature-selection robustness.|Shows consistent improvements across several datasets	Metaheuristic methods can be stochastic and sensitive to hyperparameters.|Clinical interpretability of selected features may be limited.
[34]	Weakly supervised attention multiple instances learning to predict HER2 status from H&E slides.	106 BC cases with known HER2 status.	Validation AUC = 0.92; test AUC = 0.88	Predicts a clinically relevant biomarker from routine H&E|Strong AUC on validation.	Need to be generalized on large and diverse datasets to ensure robust predictions

**Table 2:** The results for the three classes of the dataset used with YOLOv11.

Model	Precision	Recall	F1	Accuracy	Specificity	BAC	Average
YOLO11n	88.76%	88.69%	88.72%	92.81%	93.22%	90.96%	90.53%
YOLO11s	91.77%	91.88%	91.82%	94.87%	95.76%	93.82%	93.32%
YOLO11m	95.40%	95.37%	95.38%	97.05%	97.78%	96.57%	96.26%
**YOLO11l**	**95.47%**	**95.47%**	**95.47%**	**97.09%**	**97.73%**	**96.60%**	**96.30%**
YOLO11x	95.45%	95.46%	95.45%	97.06%	97.66%	96.56%	96.27%

**Table 3:** Performance comparison between YOLOv11-Large and established classification baselines.

Model	Accuracy	Precision	Recall	F1	BAC
YOLOv11-Large	97.09	95.47	95.47	95.47	96.60
ResNet-50	94.23	92.15	92.31	92.23	93.12
DenseNet-121	95.18	93.87	93.95	93.91	94.56
EfficientNet-B4	95.89	94.52	94.68	94.60	95.21
ViT-Base	94.76	92.89	93.05	92.97	93.87

**Table 4:** Performance at the Slide Level (Majority Voting).

Metric	Value
Accuracy	94.44
Precision	93.75
Recall	94.44
F1-Score	94.09
BAC	93.89

## Data Availability

The dataset is available at https://doi.org/10.5061/dryad.0cfxpnw5q. The data are released for research purposes and contain no personally identifiable information.
